# Comparison of Two Different Threshold Values for the Measurement of Gastric Residual Volume on Enteral Nutrition Support in the Neurocritically Ill Patients

**DOI:** 10.3389/fnut.2022.871715

**Published:** 2022-06-21

**Authors:** Fang Liu, Gang Liu, Rui Sun, Jinli Wang, Miao Li, Lichao Gong, Yingying Su, Yan Zhang, Yuan Wang

**Affiliations:** ^1^Department of Neurology, Xuanwu Hospital, Capital Medical University, Beijing, China; ^2^China National Brain Injury Evaluation Quality Control Center, Xuanwu Hospital, Capital Medical University, Beijing, China

**Keywords:** enteral nutrition, gastric residual volume, neurocritical care, aspiration, neurological disease

## Abstract

**Background:**

Although recommendations on gastric residual volume (GRV) have been applied to the clinical practice of patients who are intubated, evidence-based data about the GRV of patients who are neurocritically ill are still lacking. We conducted this study to investigate the safety of increased GRV in patients who are neurocritically ill on enteral nutrition (EN) support.

**Methods:**

Patients who are neurocritically ill feeding through intragastric enteral tubes were recruited consecutively between July 2018 and June 2021. Patients were divided into a control group (GRV 100 ml) and a study group (GRV 200 ml). Demographic data, admission diagnosis, and severity scores were collected from the patient medical records. The frequency of diet volume ratio (diet received/diet prescribed), the incidence of gastrointestinal complications, and outcome variables were evaluated.

**Results:**

There were 344 patients enrolled, of whom 197 had mechanical ventilation support. High GRV events in the control group were more frequent than those in the study group (38.1 vs. 22.8%, *p* = 0.002). The total gastrointestinal complication rate did not differ significantly between the two groups (study group: 61.1%, 102/167 vs. control group: 67.9%, 114/168). In the study group, two patients had aspiration (1.2 vs. 0%, *p* = 0.245). The study group showed a superior diet volume ratio, but the difference was not significant. The outcomes of the study group were slightly better than those of the control group; however, no significant differences were observed between the two groups concerning the length of stay in the neurointensive care unit (19.5 days vs. 25.3 days) and mortality (10.8 vs. 14.9%) at discharge.

**Conclusion:**

Our results suggest that 200 ml may be a safe normal limit for GRV in patients who are neurocritically ill.

## Introduction

Enteral nutrition (EN), compared with parenteral nutrition (PN), is associated with decreased infectious complications and shortened length of stay in the intensive care unit (ICU) (1–5). Recent guidelines also recommend starting EN within 24–48 h of ICU admission to reduce mortality and infectious complications (6, 7).

Gastric residual volume (GRV) is the volume of gastric fluid removed by aspirating the stomach contents with a syringe attached to a gastric tube (8). A high GRV is observed if there is delayed gastric emptying. A high volume accumulated in the stomach could result in pulmonary aspiration (9). Therefore, monitoring of gastric residual volume (GRV) is used as an indicator of diet tolerance and to prevent aspiration in patients receiving early EN in clinical practice.

However, feeding interruption is also common for various reasons, including GRV (10). Yip et al. (11) found that 38% of feeding interruptions resulted from high GRV. Gastric intolerance is the main gastrointestinal complication during EN in patients who are critically ill. Guidance of EN toward a target goal rate is usually determined by upper gastrointestinal function. The most common method of assessing this is *via* the measurement of GRV in clinical practice (12). However, recommendations on the normal limit for GRV in patients who are critically ill and treated with EN are not uniform, and volumes from 50 to 500 ml were reported in prior studies (13–15).

These recommendations have been applied to clinical practice for patients who are mechanically ventilated. In addition, many patients with neurological diseases, such as consciousness disorders caused by brain injury and dysphagia caused by stroke, need EN but not mechanical ventilation. Evidence-based data to guide GRV in patients with neurocritical illness are still lacking. In China, a GRV of 100 ml is recommended for patients who are neurocritically ill based on expert consensus (16, 17).

This study aimed to investigate the effect of increasing the limit for GRV from 100 to 200 ml for patients who are neurocritically ill with EN.

## Methods

### Study Design

This was a single-center, prospective, and observational study. We enrolled all consecutive patients who were administered with enteral nutrition *via* a feeding tube in the neurointensive care unit (NICU) of Xuanwu Hospital, Capital Medical University, between July 2018 and June 2021. Patients were included in the study if they received EN *via* a nasogastric or orogastric tube, were aged 18 years or older, and had a length of stay ≥ 24 h. Exclusion criteria included patients who received EN *via* a nasojejunal, jejunostomy, or gastrostomy tube; were pregnant; had contraindications to EN (e.g., occlusion, ileus, or gastrointestinal ischemia); or had contraindications to gastric probing (e.g., esophageal stenosis, surgery, recent gastroesophageal trauma, and hematemesis). They were divided into two groups: the control group (GRV 100 ml) and the study group (GRV 200 ml) according to a table of the random digit.

This study was approved by the Ethics Committee of Xuanwu Hospital, Capital Medical University, and is adhered to the Declaration of Helsinki. Written informed consent was obtained from the patients or their guardians.

### Medical Care

A working group including physicians, nurses, and dietitians reviewed the feeding protocol following the recommendations of the Chinese Nutritional Working Group (16).

All patients received EN *via* a 14-Fr nasogastric tube. The intragastric position of tube feeding was radiographically confirmed before diet infusion. EN was delivered continuously *via* a feeding pump in both groups. The goal EN for the two groups was calculated according to the patient’s body weight, with 1 ml of formula per kg of body weight being administered if the BMI was ≤ 30 kg/m^2^, or 1 ml of formula per kg of ideal body weight if the body mass index (BMI) was > 30 kg/m^2^. The optimal calorie supply was assumed to be 25 Kcal/kg/day. Patients were kept in a semi-recumbent position with the head of the bed elevated to 30°. The initial infusion rate was 25 ml/h, which was then gradually increased to the optimal infusion rate.

**FIGURE 1:**
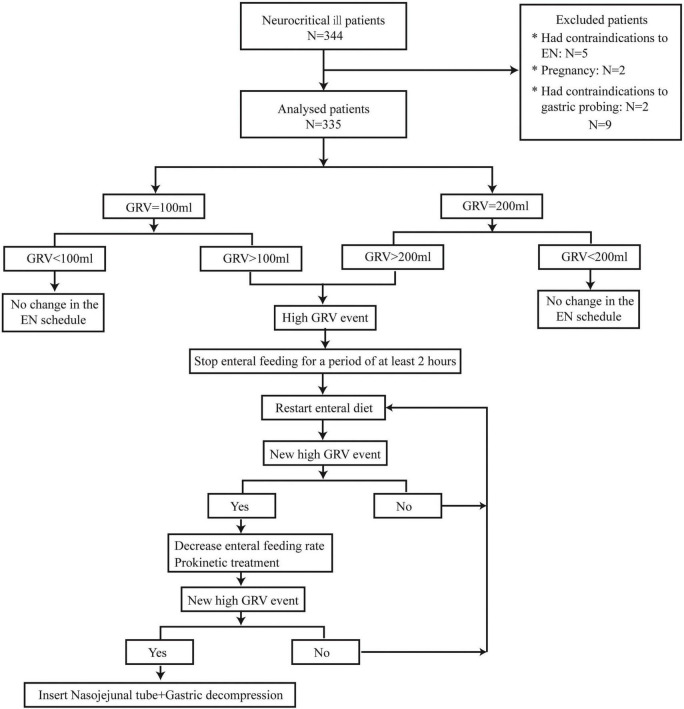
The study flow chart.

We used the syringe aspiration method with a 50-ml syringe to measure the GRV every 4 h. If 50 mL was withdrawn into the syringe, the fluid was emptied into a calibrated container, and the procedure was repeated until no more fluid could be withdrawn. Any amount less than the threshold value was reinstilled into the feeding tube. The tube was then flushed with 30 mL of water before being reconnected to the feeding pump. If a high GRV event occurred, EN could be interrupted for at least 2 h to promote gastric rest before restarting EN. If the GRV remained above the threshold, prokinetic treatment was administered, and the EN infusion rate was reduced. Insertion of a nasojejunal tube was performed in cases of repeated events of high GRV (Figure 1). Parenteral nutrition (PN) was used to complement nutritional requirements in cases of underfeeding with EN.

### Clinical Data Collection

Demographic data, admission diagnosis, Glasgow Coma Scale (GCS) score, acute physiologic and chronic health evaluation (APACHE II) score, and sequential organ failure assessment (SOFA) score were collected from the patients’ medical records. The NICU and hospital lengths of stay and mortality rates were also recorded. High GRV was defined as a GRV equal to or greater than 100 ml (control group) or 200 ml (study group). Tolerability, referring to gastrointestinal complications, was defined as follows: abdominal distension (abdominal changes on daily physical exam with tympany and/or absence of bowel sounds), high gastric residuals (GRV ≥ threshold value), vomiting (enteral formula ejected through the mouth), astriction (dry stool and frequency less than 3 times a week), diarrhea (five or more liquid stools in a 24-h period or an estimated stool volume equal to or greater than 2,000 ml/day), and pulmonary aspiration (feed was detected in the tracheal aspirate). Calorie intake was calculated as follows: diet volume ratio = (administered volume of diet/prescribed volume) × 100%.

### Statistical Analysis

All data were analyzed with SPSS, version 26.0 (SPSS Inc., Chicago, IL, United States). Categorical variables between the two groups were compared using chi-squared tests. A Pearson χ*^2^* test was used when no subgroup had an expected count below 5; otherwise, Fisher’s exact test was performed. A two-tailed *t-*test for normally distributed continuous variables was performed. The Mann–Whitney U test was used in cases where the variable was not normally distributed. Odds ratios (ORs) and 95% confidence intervals (95% CIs) were calculated. *P*-values of less than 0.05 were considered statistically significant.

## Results

### Population

A total of 344 patients were enrolled in the NICU, and 9 patients were excluded from the final analysis for the following reasons: five had contraindications to EN, two were pregnant, and two had contraindications to gastric probing. Of the 335 included patients, 167 were in the study group, and 168 were in the control group. The patients were aged between 18 and 78 years, with a mean age of 57.1 years (SD 17.2). The primary diagnosis at admission was cerebrovascular disease (*n* = 188, 56.1%), followed by central nervous system (CNS) infection (*n* = 65, 19.4%), CNS immune disease (*n* = 44, 13.1%), Guillain–Barre syndrome (GBS, *n* = 11, 3.3%), myasthenia gravis (MG, *n* = 9, 2.7%), neuromuscular disease (*n* = 9, 2.7%), and others (*n* = 9, 2.7%). A total of 197 patients (58.8%) were on mechanical ventilation support (control group: 97, study group: 100).

There were no significant differences between the two groups in terms of age, sex, BMI, GCS score, APACHE II score, SOFA score, or diagnosis at admission. The baseline characteristics of both groups are shown in Table 1.

**TABLE 1:** Characteristics of patients in two groups.

Characteristic	Control group, *N* = 168 (GRV 100 ml)	Study group, *N* = 167 (GRV 200 ml)	*P*-value
Age, years^a^	56.8 ± 16.65	57.4 ± 20.24	0.88
Gender			
Male/Female	87/81	89/78	0.78
GCS score^a^	9.2 ± 3.1	9.3 ± 3.4	0.58
APACHE II score^a^	23.6 ± 7.1	23.2 ± 6.8	0.81
SOFA score^a^	8.2 ± 4.3	7.9 ± 3.9	0.72
Mechanical ventilation support (%)	97 (57.7%)	100 (59.9%)	0.69
BMI^a^	24.37 ± 3.97	24.20 ± 4.26	0.83
Diagnosis at NICU admission			0.81
Cerebrovascular disease (%)	92 (54.8%)	96 (57.5%)	
CNS infection (%)	36 (21.4%)	29 (17.4%)	
CNS immune disease (%)	21 (12.5%)	23 (13.8%)	
MG (%)	6 (3.6%)	3 (1.8%)	
Neuromuscular disease (%)	5 (3.0%)	4 (2.4%)	
GBS (%)	4 (2.4%)	7 (4.2%)	
Others (%)^b^	4 (2.4%)	5 (3.0%)	

*APACHE II, acute physiologic and chronic health evaluation score; BMI, body mass index; CNS, central nervous system; GCS, Glasgow coma scale; GRV, gastric residual volume; IQR, interquartile range; SOFA, sequential organ failure assessment.*

*^a^Mean ± SD, ^b^Control group: 2 patients with multiple system atrophy, 1 patient with Parkinson’s disease and 1 patient with tumor; Study group: 3 patients with hypoxic encephalopathy and 2 patients with traumatic brain injury.*

### Tolerability

**FIGURE 2:**
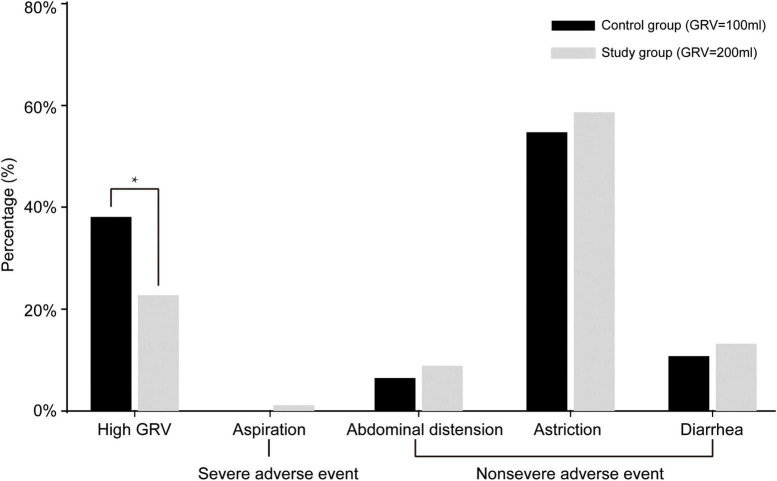
Gastrointestinal complications of patients in the two groups. In the study group, 38 patients (22.8%) had high gastric residual volume (GRV) events, 2 patients (1.2%) had aspiration, 15 patients (8.9%) had abdominal distension, 98 patients (58.7%) had astriction, and 24 (14.4%) had diarrhea. No patients in the control group had severe adverse events (aspiration); in this group, 64 patients (38.1%) had high GRV events, 11 patients (6.5%) had abdominal distension, 92 patients (54.8%) had astriction, and 19 (11.3%) had diarrhea. *P < 0.05.

Although there were more severe adverse events (aspiration) in the study group than in the control group, there was no significant difference (1.2 vs. 0%, χ*^2^* **=** 1.012, OR **=** 0.047, 95% CI = 0.995–1.029, *p* = 0.248). The overall gastrointestinal complication rates were 61.1% (102/167) in the study group and 67.9% (114/168) in the control group (χ*^2^* **=** 1.681, OR **=** 0.743, 95% CI = 0.474–1.165, *P* = 0.195). The difference in the frequency of high GRV was statistically significant, 22.8% (38/167) vs. 38.1% (64/168) for the study and control groups, respectively (χ*^2^* **=** 9.307, OR **=** 0.047, 95% CI = 0.297–0.772, *P* = 0.002). There were no significant differences between the study group (GRV 200 ml) and control group (GRV 100 ml) with respect to abdominal distension (8.9 vs. 6.5%, χ*^2^* **=** 0.693, OR **=** 1.027, 95% CI = 0.965–1.093, *p* = 0.405), astriction (58.7 vs. 54.8%, χ*^2^* **=** 0.524, OR **=** 1.095, 95% CI = 0.856–1.400, *p* = 0.469), or diarrhea (14.4 vs. 11.3%, χ*^2^* **=** 0.702, OR **=** 1.271, 95% CI = 0.724–2.230, *p* = 0.402) (Figure 2).

### Calorie Intake-Diet Volume Ratio

The study group had superior mean ratios to the control group at week 1 (88.6 vs. 83.4%), week 2 (89.1 vs. 84.9%), and week 3 (93.2 vs. 90.5%) without significant differences. A total of 45 patients could not complete 3 weeks of EN for the following reasons: death within 3 weeks (study group: 18 vs. control group: 25, *P* = 0.262) and EN transition to oral feeding (study group: 10 vs. control group: 9, *P* = 0.818).

### Outcomes

The study group showed better outcomes at discharge than the control group, but these differences did not reach significance. The mortality (GRV 200 ml vs. GRV 100 ml) rate was 10.8% (18/167) vs. 14.9% (25/168) at discharge (χ*^2^* **=** 1.260, OR **=** 0.724, 95% CI = 0.411–1.277, *p* = 0.262). The median NICU length of stay was 19.5 days vs. 25.3 days (*P* > 0.05) for the study and control groups, respectively.

## Discussion

In the past two decades, several organizations have issued clinical practice guidelines and recommendations regarding GRV measurements (6, 7, 18–20). Following the recommendations, interruption of EN should be avoided when the amount of GRV is below 500 ml in units, where the intervention still needs to be performed (quality of evidence: low) (6). As such, routine GRV measurement in the ICU is not recommended by the most recent guidelines, as the evidence underpinning this recommendation is controversial. Several guidelines, studies, and organizations, including the American Society for Parenteral and Enteral Nutrition, currently recommend that the use of GRVs should be abandoned as part of standard care in the ICU.

The clinical value of GRV in the evaluation of enteral feeding tube tolerance has been controversial. Monitoring of GRV is recommended to prevent ventilator-associated pneumonia (VAP) in patients receiving early EN (6, 18, 21). Some studies have recommended that nutritional support should be modified above GRV values of 100–500 ml (5, 22, 23). However, the reliability and effectiveness of measuring GRV during EN have been seriously challenged (24). For patients who are mechanically ventilated, monitoring of GRV appears unnecessary to guide nutrition. It was found that not monitoring GRV did not increase the feeding intolerance rates, ventilator-associated pneumonia rates, or mortality rates (15, 25, 26). Therefore, the main reasons to abandon GRV monitoring were as follows: a concept of not monitoring GRV could thereby reduce nurses’ workload, and monitoring GRV could not significantly improve patient outcomes. However, in these studies, the study population was mainly patients with mechanical ventilation.

In clinical practice, ICU nurses still perform GRV measurements in patients receiving EN. GRV is one surrogate parameter indicating disorders of gastrointestinal motility. A regular clinical evaluation of the abdomen (clinical examination, radiologic examination in selected cases, and frequency of bowel movements) is also a part of the monitoring process. The GRV measurement is especially important for staff with less experience and training (27).

A GRV of 100 ml is recommended for patients who are neurocritically ill (16, 17). In this population, EN is often provided to patients who are not on mechanical ventilation. Patients with neurocritical illness who have consciousness disturbances or dysphagia also require EN supplementation. The reason for the low GRV in severe neurological patients is that they are more susceptible to accidental inhalation, and adverse reactions are not easy to observe. In our study, 2 patients (1.2%) had aspiration in the study group. We found that a high GRV (200 ml) did not significantly increase the incidence of this severe adverse event. In addition, there were no other gastrointestinal complications.

According to a previous study, increasing the limit of GRV can be considered a measure to decrease the energy deficit (28). In our study, patients with a high GRV also received a more enteral diet and had a superior calorie intake ratio (week 1, 88.6 vs. 83.4%; week 2, 89.1 vs. 84.9%; and week 3, 93.2 vs. 90.5%). Although the differences did not reach significance, the study group showed a shorter ICU length of stay and lower mortality at discharge than the control group. More research is still needed to confirm the effect of increasing GRV on the efficacy of diet administration among patients who are critically ill receiving EN.

This study also had some limitations. The study was conducted in a single-center, and the nature of the intervention did not allow blinding. The etiological classification was not taken into consideration in this study. Although a high GRV appeared to be well-tolerated in this study, careful bedside evaluation of risk should be performed, especially for patients who are comatose.

## Conclusion

In this study, we investigated the effects of increasing the limit for GRV from 100 ml up to 200 ml in patients who are neurocritically ill on EN. We found that the limit of 200 ml did not result in more adverse effects. Therefore, the limit of 200 ml could be recommended as a normal limit for GRV of patients who have neurocritical illness.

## Data Availability Statement

The raw data supporting the conclusions of this article will be made available by the authors, without undue reservation.

## Ethics Statement

The study was approved by the Ethics Committee of Xuanwu Hospital, Capital Medical University, and it adhered to the Declaration of Helsinki. Written informed consent was obtained from patients or their guardians.

## Author Contributions

FL, GL, and YW contributed to the conception and designed the study. FL, GL, RS, JW, ML, and LG contributed to the acquisition and analysis of data. FL, GL, YS, YZ, and YW contributed to drafting the manuscript and figures. All authors contributed to the article and approved the submitted version.

## Conflict of Interest

The authors declare that the research was conducted in the absence of any commercial or financial relationships that could be construed as a potential conflict of interest.

## Publisher’s Note

All claims expressed in this article are solely those of the authors and do not necessarily represent those of their affiliated organizations, or those of the publisher, the editors and the reviewers. Any product that may be evaluated in this article, or claim that may be made by its manufacturer, is not guaranteed or endorsed by the publisher.
